# Comparison of the JNC7 and 2017 American College of Cardiology/American Heart Association Guidelines for the Management of Hypertension in Koreans: Analysis of Two Independent Nationwide Population-Based Studies

**DOI:** 10.3390/ijerph16245134

**Published:** 2019-12-16

**Authors:** Won-Jun Choi, Hye-Sun Lee, Jung Hwa Hong, Hyuk-Jae Chang, Ji-Won Lee

**Affiliations:** 1 Family Medicine, Graduate School, Yonsei University College of Medicine, Seoul 03722, Korea; slashwj@gmail.com; 2 Biostatistics Collaboration Unit, Yonsei University College of Medicine, Seoul 06273, Korea; HSLEE1@yuhs.ac; 3 Institute of Health Insurance & Clinical Research, National Health Insurance Service Ilsan Hospital, Seoul 10444, Korea; jh_hong@nhimc.or.kr; 4 Division of Cardiology, Severance Cardiovascular Hospital, Yonsei University College of Medicine, Yonsei University Health System, Seoul 03722, Korea; 5 Department of Family Medicine, Gangnam Severance Hospital, Yonsei University College of Medicine, Seoul 06273, Korea

**Keywords:** hypertension, mortality, guidelines, blood pressure

## Abstract

The optimal blood pressure (BP) guidelines in Asian populations have not been determined. We compared all-cause and cardiovascular mortality based on the Joint National Committee 7 (JNC7) and 2017 American College of Cardiology/American Heart Association (ACC/AHA) guidelines. The National Health Insurance System-National Health Screening Cohort (NHIS-HEALS) and Korea National Health and Nutrition Examination Survey (KNHANES) were utilized. BPs were classified into three groups according to each guideline, and survival rates were analyzed with Kaplan-Meier curves and log-rank tests. Hazard ratios (HRs) were calculated using multivariable cox regression analyses, and the discriminatory ability for clinical outcomes was assessed by Harrell’s C-indexes. The JNC7 guidelines demonstrated a linear association between BP levels and survival outcomes. Adjusted HRs from the JNC7 guidelines differentiated the hypertension group (≥140/90) from the pre (130/80–139/89) and normal (<130 and <80) BP groups in clinical outcomes. In contrast, the 2017 ACC/AHA guidelines showed inconsistent survival outcomes according to BP classification (normal: <120 and <80, elevated: 120–129, and <80, and HTN: ≥130/80). According to Harrell’s C-indexes, the JNC7 guidelines had greater discrimination ability in survival outcomes in the NHIS-HEALS dataset. Our results suggest that the JNC7 guidelines are more appropriate than the 2017 ACC/AHA guidelines in Korean populations.

## 1. Introduction

In 2017, the American College of Cardiology/American Heart Association (ACC/AHA) published new blood pressure (BP) management guidelines that included changing the diagnostic standard for hypertension from a starting BP of 140/90 mm Hg, as in the Joint National Committee 7 (JNC7) guidelines, to a BP of 130/80 mm Hg. Additionally, the 2017 ACC/AHA guidelines recommend antihypertensive medication for adults at high risk of cardiovascular disease (CVD) with systolic BP (SBP) 130 to 139 mm Hg or diastolic BP (DBP) 80 to 89 mm Hg as well as treatment to lower SBP/DBP to <130/80 mm Hg for all adults taking antihypertensive medication [1].

The systolic blood pressure intervention trial (SPRINT) and recent meta-analysis revealed an intensive BP reduction with antihypertensive treatment was beneficial in reducing cardiovascular outcomes according to the new ACC/AHA guidelines [2,3]. Subsequent studies supported the appropriateness of the new guidelines and have shown a linear association between BP levels, all-cause mortality, and the risk of CVD with the lowest risk at a SBP level less than 130 mm Hg [4,5].

In contrast, several studies, including the valsartan in elderly isolated systolic hypertension (VALISH) and action to control cardiovascular risk in diabetes (ACCORD) studies, failed to demonstrate the benefit of the new BP guidelines [6,7] and inconsistent findings make it difficult to broadly apply the 2017 ACC/AHA guidelines. Indeed, the 2018 European guidelines for the management of hypertension maintained the existing hypertension definition of 140/90 mm Hg [8].

Guidelines for hypertension need to consider the disparity of country- or race-specific risk factors. BP related CVDs are more common in Asians than in Westerners [9]. However, it remains unknown whether intensive BP reduction leads to improved all-cause mortality and cardiovascular outcomes. Moreover, the optimal guidelines for discriminating high risk groups in terms of hypertension in Asian populations have not been determined.

Therefore, we aimed to compare all-cause and CVD mortality in a Korean population according to the JNC7 and 2017 ACC/AHA guidelines using two large nationally representative databases.

## 2. Materials and Methods

### 2.1. Study Population and Data Collection

#### 2.1.1. National Health Insurance System-National Health Screening Cohort (2006–2015 NHIS-HEALS)

This study was based on data obtained from the NHIS-HEALS, a nationally retrospective cohort study conducted by the Korea Centers for Disease Control and Prevention. The NHIS is a universal health coverage program, and all insured individuals and their dependents are required to undergo general health examinations every 2 years. The National Health Examination followed standardized procedures, and its validity is described elsewhere [10].

**Figure 1:**
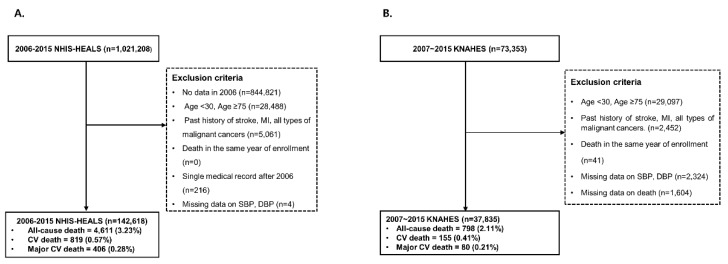
Flow chart of the study population. **A**: 2006–2015 NHIS-HEALS; **B**: 2007–2015 KNAHES.

Study populations were followed from 1 January 2006 until the date of a cardiovascular event, death, or 31 December 2015, whichever came first. We extracted 1,021,208 participants aged 30–74 years whose data were available and excluded individuals who met any of the following criteria (n = 878,590): Younger than 30 or older than 75 years of a age; history of hospitalization for a diagnosis of myocardial infarction (MI; Korean Standard Classification of Diseases, KCD codes I21–I23) or stroke (KCD codes I60–I64); any type of malignant cancer; death in the year of enrolment; single medical record after 2006; and those with missing SBP, DBP, or death data. Following these exclusions 142,618 participants were included in the final analysis as shown in Figure 1.

Self-reported cigarette smoking and alcohol consumption were determined by questionnaire. Each participant was categorized as a non-smoker, ex-smoker, or current smoker with respect to smoking status. Participants were categorized as non-drinkers, intermittent drinkers (≤3–4 times a week), or daily drinkers with respect to alcohol use. Physical activity was divided into five groups according to the amount of exercise per week, and household income was divided into five groups based on the 10th quantile information.

BP was measured at local hospitals, each of which met the internal and external quality control procedures of the Korean Association of External Quality Assessment Service. After participants rested while seated for at least 2 min, and BP measurements were taken by digital or automatic monitors during the health examination. All BP measurements, including BP data before the index period, were used to calculate mean BP.

#### 2.1.2. Korea National Health and Nutrition Examination Survey (2007–2015 KNHANES Cohort)

We obtained data from the 2007–2015 Korean National Health and Nutrition Examination Survey (KNHANES) cohort, a nationally representative survey conducted by the Korean Ministry of Health and Welfare. KNHANES datasets have been provided publicly, 10 but recent datasets have been further matched with death information from any cause. As a result, the data can be used for nationally representative death statistics. Study populations were followed from 1 January 2007 to 31 December 2015.

We extracted 73,353 participants aged 30–74 years and excluded participants who met any of the following criteria (n = 35,518): Participants younger than 30 or older than 75 years, a history of stroke, acute MI, or any type of malignant cancer, death in the year of enrollment, and those with missing SBP, DBP, or death data. Following these exclusions 37,835 participants were included in the final analysis as shown in Figure 1.

Self-reported cigarette smoking and alcohol consumption were determined by questionnaire. Each participant was categorized as a non-smoker, ex-smoker, or current smoker with respect to smoking status. Participants were also asked about the frequency of their alcohol intake and weekly physical activity. Alcohol use was defined if one of two criteria was satisfied: Drinking quantity (≥7 drinks for males and ≥5 drinks for females) and frequency (≥2 times per week).

BP measurements were taken at local examination centers by trained examiners. BP was measured three times, and the average of the second and third measurements was used for analysis [10].

### 2.2. Outcome Measurement

The primary outcomes of the study were all-cause mortality and all cardiovascular mortality. All cardiovascular mortality was defined as death from a disease of the circulatory system (KCD codes I00–I99). We selected myocardial infarction (MI, KCD codes I21–I23), hemorrhagic stroke (KCD codes I60–I62), and ischemic stroke (KCD code I63) for inclusion among the detailed causes of cardiovascular mortality.

### 2.3. Statistical Analysis

BPs were classified into three groups according to each guideline. Under the JNC7 guidelines, BPs were categorized as follows: normal (<130 and <80), pre-hypertension (130/80–139/89), and hypertension (HTN, ≥140/90). Under the 2017 ACC/AHA guidelines, BPs were classified as normal (<120 and <80), elevated (120–129 and <80), and HTN (≥130/80). The characteristics of the study population were presented as means ± standard deviations and frequencies (percentages). Groups were compared using one-way analysis of variance (ANOVA) for continuous variables and chi-square tests for categorical variables.

The survival rates of each group according to the adjustment criteria presented in each guideline were analyzed by Kaplan-Meier curves and log-rank tests. The warranty period was defined as the time required for the cumulative mortality incidence to reach 0.5% for each group. If the 0.5% threshold was not met, the value was expressed as the time of the last follow-up. In addition, incidence per 1000 person-years was calculated for each group. The hazard ratios (HRs), 95% confidence intervals (CIs), and *p* values for trends with reference to the pre-HTN group (130/80–139/89) in the JNC7 guidelines and the elevated BP group (120–129 and <80) in the 2017 ACC/AHA guidelines were calculated using multivariable Cox regression analyses after adjusting for age, sex, and body mass index (BMI) in the KNHANES cohort and age, sex, BMI, physical activity, household income, smoking status, alcohol status, fasting serum glucose, and total cholesterol in the NHIS-HEALS cohort. The KNAHES cohort had a smaller number of events compared to the NHIS-HEALS cohort. Therefore, we adjusted the KNAHES cohort for the smallest exploratory variable, while the NHIS-HEALS cohort was adjusted for additional variables. To evaluate the predictability of the JNC7 and 2017 ACC/AHA guidelines, we calculated Harrell’s c-index (95% CI). To calculate the 95% CIs and *p*-values for Harrell’s C-index and the differences between JNC7 and ACC/AHA, we used a bootstrap method and resampled 1000 times.

The study was approved by the Institutional Review Board of Yonsei University Health System (IRB number: 3-2018-0160), and the requirement for informed consent was waived. All statistical analyses were performed using SAS software, version 9.4 (SAS Institute Inc., Cary, NC, USA). All statistical tests were two-sided, and statistical significance was determined at *p* < 0.05.

## 3. Results

Using the NHIS-HEALS and KNAHES datasets of 1,021,208 and 73,353 individuals, respectively, we identified 142,618 and 37,835 adults for inclusion in this study. The number of all-cause mortality (ACM), all cardiovascular deaths (ACD), and major cardiovascular deaths (MACE; acute MI, ischemic stroke, and hemorrhagic stroke) in each dataset are shown in Figure 1. During follow-up, 4611 ACM and 819 CVD events occurred in the NHIS-HEALS cohort, and 789 ACM and 155 CVD events occurred in the KNAHES cohort. Table 1 presents the baseline characteristics according to BP levels under the two different criteria. The prevalence of hypertension was estimated to be 40.64% (NHIS-HEALS) and 44.8% (KNAHES) based on the 2017 ACC/AHA guidelines, which was a dramatic increase compared to the 9.64% (NHIS-HEALS) and 18.4% (KNAHES) prevalence rates based on the JNC7 guidelines. Individuals with a higher BP tended to be older, consumed more alcohol, had a higher BMI, and had higher fasting serum glucose levels in both cohorts.

**Figure 2:**
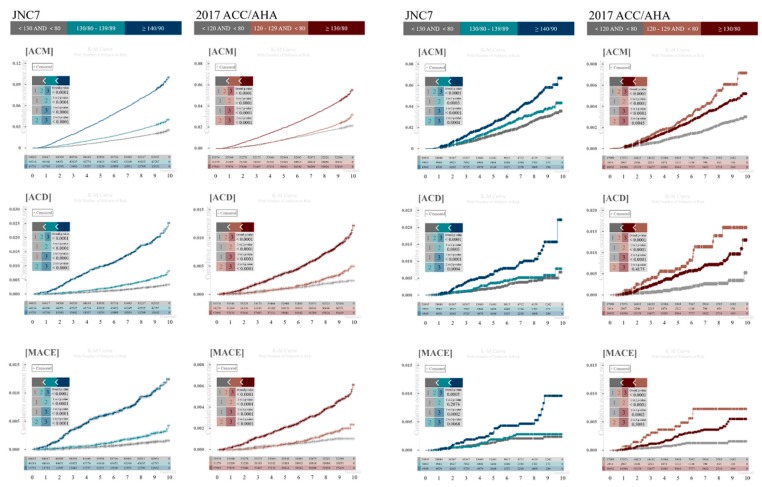
Kaplan-Meier curves with log-rank tests for the cumulative incidence of for all-cause mortality, all cardiovascular death, and major cardiovascular death according to JNC7 and 2017 ACC/AHA guidelines.

Figure 2 shows the cumulative incidence of the three outcomes (ACM, ACD, and MACE) for each guideline using Kaplan-Meier curves and log-rank tests. Based on the JNC7 guidelines, there was a significant linear trend towards an increased risk of all three outcomes with worse BP control regardless of dataset. The 2017 ACC/AHA guidelines, however, showed different results. In the NHIS-HEALS dataset, the 2017 ACC/AHA guidelines showed increased incidence of all three outcomes in sequential BP groups, but in the KNAHES dataset, the cumulative incidence of ACM, ACD, and MACE was highest in the elevated BP group, followed by the HTN and normal BP groups.

The warranty periods were defined as the duration in years that the cumulative mortality rate remained <0.5%. Based on the JNC7 guidelines, the HTN group had an unfavorable warranty period in all three outcomes compared to the elevated BP and normal BP groups in the two datasets. However, according to the 2017 ACC/AHA guidelines, results were inconsistent between the NHIS-HEALS and KNAHES datasets. Although the warranty periods for ACM and ACD were shortest in the HTN group in the NHIS-HEALS dataset, the elevated BP group had the shortest warranty period for ACM and ACD in the KNAHES dataset. Based on the JNC7 guidelines, the event rates for ACM, ACD, and MACE per 1000 person-years increased linearly with increasing BP in both datasets. However, according to the 2017 ACC/AHA guidelines, the cumulative incidence of ACM, ACD, and MACE was highest in the elevated BP group, followed by the HTN and normal BP groups in the KNAHES dataset. In other words, the 2017 ACC/AHA guidelines did not show linearity in warranty periods and event rates per 1000 person-years Table 2.

Table 3 shows adjusted HRs for ACM, ACD, and MACE according to each guideline after adjusting for all variables. Based on the JNC7 guidelines, the hazard ratio (95% CIs) was statistically significantly higher in the HTN group in all three outcomes compared to the other two groups in both datasets: ACM 1.84 (1.67–2.00), ACD 2.72 (2.26–3.27), and MACE 3.25 (2.51–4.21) in the NHIS-HEALS dataset and ACM 1.24 (1.05–1.48), ACD 1.83 (1.26–2.65), and MACE 1.73 (1.04–2.90) in the KNAHES dataset. Unlike the JNC7 guidelines, the 2017 ACC/AHA guidelines showed different results between the two datasets. The HTN group showed the highest hazard ratio in all three outcomes in the NHIS-HEALS dataset: ACM 1.15 (1.06–1.24), ACD 1.76 (1.42–2.17), and MACE 2.09 (1.53–2.84). However, there were no statistically significant differences between the normal group and the elevated BP group for ACD or MACE in the NHIS-HEALS dataset. Conversely, in the KNAHES dataset, the hazard ratio for ACM was significantly higher in the normal group than in the elevated BP group according to the 2017 ACC/AHA guidelines. Moreover, based on the 2017 ACC/AHA guidelines, there was no statistically significant difference for all three HRs between the elevated BP group and the HTN group in the KNAHES dataset. Instead, the normal group had the lowest HRs for all three outcomes in the KNAHES dataset.

To evaluate the potential discriminatory ability of the two guidelines for clinical outcomes, Harrell’s C-indexes were calculated and are presented in Table 4. These results suggest that the JNC7 guidelines have a greater discrimination ability in the NHIS-HEALS dataset and show no significant difference between the two guidelines in the KNAHES dataset.

## 4. Discussion

Hypertension is a major risk factor for cardiovascular morbidity and mortality [11]. Due to the association of hypertension and BP, optimal BP thresholds and treatment goals have long been a source of debate. Although the recent 2017 ACC/AHA guidelines recommend treating patients to reduce SBP/DBP to <130/80 mm Hg [1], it is still uncertain whether such aggressive BP control results in improved clinical outcomes. Moreover, there is little direct evidence to guide the choice of target BP in Asian populations.

To address these unanswered questions, we compared clinical outcomes using the JNC7 and 2017 ACC/AHA guidelines in two nationally representative Korean population datasets. Based on the JNC7 guidelines, there was a linear association between BP levels and survival outcomes in both datasets. Similarly, the HTN group had unfavorable survival outcomes as reflected by warranty periods and event rates per 1000 person-years, regardless of dataset. In addition, adjusted HRs calculated using the JNC7 guidelines were able to differentiate the HTN group from the elevated and normal BP groups in all clinical outcomes. In contrast, the 2017 ACC/AHA guidelines showed inconsistent survival outcomes according to BP classification in the two datasets. Moreover, the JNC7 guidelines had more discrimination ability for all survival outcomes than the 2017 ACC/AHA guidelines according to Harrell’s c-indexes in the NHIS-HEALS dataset.

It is uncertain why our results differ from the SPRINT study and recent meta-analyses [12] with emphasis on the strict BP lowering treatment. The differences may be explained in part by country- or ethnicity-specific factors and differing health environments. For example, the cutoff value for the definition of obesity is lower for Asian populations than for Western populations [13]. Asian populations tend to have a high salt intake, salt sensitivity, masked HTN, and nocturnal HTN [13]. Also, exaggerated morning BP surges are more frequent in Asian populations compared with Western populations [13]. Therefore, the influence of BP on health outcomes may differ among Asians and Westerners [14]. Future studies are warranted to clarify the possible mechanisms underlying the differences related to race and ethnicity.

Additionally, several studies in Western populations demonstrated inconsistencies with the SPRINT study. The SHEP (Systolic Hypertension in the Elderly Program) trial postulated that less intensive BP control may be more suitable for older patients, [15] and a pooled analysis of the ONTARGET and TRANSCEND trials suggested that excessive reduction of BP (SBP <120 mm Hg) increased mortality and cardiovascular events compared to SBP between 120 and 140 mm Hg [16]. Furthermore, the post hoc analysis of the SPRINT trial found that even those with a lower baseline CVD risk of <18.2% experienced more harm than benefit following strict BP lowering treatment [17]. The strict BP thresholds of the new guidelines may be more appropriate for use in younger patients and in patients with higher CVD risk than those of the JNC7 guidelines. This is supported by recent epidemiologic data from the NHIS-HEALS cohort in young hypertensive patients as well as in patients with atrial fibrillation and HTN. Among young Korean adults with hypertension defined by the 2017 ACC/AHA guidelines, the risk of subsequent cardiovascular disease events was elevated compared to those with normal BP, [18] and BP between 120 and 129/<80 mm Hg was found to be the optimal BP treatment target for patients with atrial fibrillation undergoing hypertension treatment [19]. Therefore, it is reasonable that the benefits of intensive BP reduction according to the new guidelines may differ according to age and associated cardiovascular risks. In our study, we used two independent nationwide cohort databases, which enables a high degree of generalization of our results. The JNC7 guidelines showed consistent results and a linear association between BP level and survival outcome; however, the 2017 ACC/AHA guidelines failed to find a linear relationship between all-cause or cardiovascular mortality and BP classifications in the two datasets.

This study has several limitations. First, although BP measurement equipment in all health examination institutions received quality assessment according to the Basic Act on National Health Examination, the lack of device uniformity and single visit measurements may have introduced some variability into our results. Unfortunately, it was impossible to measure home BP monitoring and ambulatory BP monitoring in routine evaluations. Second, because the current study was observational, confounding factors could result in over- or under-estimation of the effect of BP on clinical outcomes. Third, we did not account for antihypertensive medications that may affect the all-cause or cardiovascular mortality and could not conclude whether those who took antihypertensive medications following the JNC7 had a significantly lower mortality than those following the 2017 ACC/AHA guidelines. Also, we did not consider the mortality of heart failure that could create structural modification of the left ventricle [20]. Fourth, there is a possibility of sampling bias as our results were not weighted due to a lack of information on sampling weights in the data obtained from the NHIS-HEALS and KNHANES. Finally, our study population was composed of only Korean adults, so the results may not be generalizable to other races or ethnicities. Further investigations are required to evaluate and select the optimal BP guidelines and to determine the applicability of a new BP threshold with consideration given to the differences in healthcare environments.

Nevertheless, our results represent real word data and reflect practical clinical conditions. To our knowledge, this is the first study to directly compare the two guidelines in terms of all-cause and cardiovascular mortality.

## 5. Conclusions

Unlike the 2017 ACC/AHA guidelines, the JNC7 guidelines demonstrated a linear association between BP levels and survival outcomes in two nationwide datasets as well as good discrimination ability in all survival outcomes. Collectively, our results suggest that the JNC7 guidelines are more appropriate than the 2017 ACC/AHA guidelines in Korean populations.

## Figures and Tables

**Table 1:** Baseline characteristics according to JNC7 and 2017 ACC/AHA guidelines.

Guidelines	JNC7 Guidelines				2017 ACC/AHA Guidelines			
**2006–2015 NHIS-HEALS**	**<130 and <80 (*n* = 84,653)**	**130/80–139/89 (*n* = 44,214)**	**≥140/90 (*n* = 13,751)**	***p*-Value ^1^**	**<120 and <80 (*n* = 53,374)**	**120–129 and <80 (*n* = 31,279)**	**≥130/80 (*n* = 57,965)**	***p*-Value ^1^**
Age	45.53 ± 10.52	49.36 ± 11.26	53.92 ± 11.94	<0.0001	44.04 ± 9.7	48.09 ± 11.35	50.45 ± 11.58	<0.0001
Female Sex, N (%)	38948 (46.01)	14,321 (32.39)	5023 (36.53)	<0.0001	27,377 (51.29)	11571 (36.99)	19344 (33.37)	<0.0001
Height, m^2^	163.76 ± 8.81	164.63 ± 9.15	162.85 ± 9.38	<0.0001	163.42 ± 8.59	164.35 ± 9.15	164.21 ± 9.24	<0.0001
Weight, kg	62.46 ± 10.46	67.11 ± 11.21	67.14 ± 11.94	<0.0001	60.87 ± 10.04	65.17 ± 10.59	67.11 ± 11.39	<0.0001
BMI, kg/m^2^	23.21 ± 2.84	24.67 ± 2.98	25.21 ± 3.24	<0.0001	22.71 ± 2.74	24.05 ± 2.81	24.8 ± 3.05	<0.0001
Physical activity, N (%)				<0.0001				<0.0001
0	43,192 (52.57)	20,980 (48.83)	6884 (51.54)		28,066 (54.2)	15126 (49.79)	27,864 (49.47)	
1–2	23,590 (28.71)	13,070 (30.42)	3533 (26.45)		14,606 (28.21)	8984 (29.57)	16,603 (29.48)	
3–4	9530 (11.6)	5235 (12.18)	1504 (11.26)		5856 (11.31)	3674 (12.09)	6739 (11.97)	
5–6	2141(2.61)	1150 (2.68)	380 (2.85)		1254 (2.42)	887 (2.92)	1530 (2.72)	
Almost everyday	3708(4.51)	2531 (5.89)	1055 (7.9)		2001 (3.86)	1707 (5.62)	3586 (6.37)	
Household income, N (%)				<0.0001				<0.0001
1–2	11,982 (14.41)	6366 (14.66)	2279 (16.74)		7450 (14.18)	4532 (14.79)	8645 (15.16)	
3–4	11,231 (13.5)	5834 (13.44)	2056 (15.1)		7084 (13.49)	4147 (13.53)	7890 (13.84)	
5–6	13,664 (16.43)	7278 (16.77)	2438 (17.91)		8407 (16.01)	5257 (17.16)	9716 (17.04)	
7–8	22,734 (27.34)	11,374 (26.2)	3340 (24.53)		14,441 (27.49)	8293 (27.07)	14,714 (25.8)	
9–10	23,555 (28.32)	12,559 (28.93)	3503 (25.73)		15,143 (28.83)	8412 (27.45)	16,062 (28.17)	
Smokers, N (%)	27,345 (33.27)	17,041 (39.69)	4827 (36.24)	<0.0001	15,827 (30.55)	11518 (37.92)	21868(38.87)	<0.0001
Alcohol drinkers, N (%)								
Non-drinker	45,514 (54.86)	20,411 (47.07)	6732 (50.1)		29,806 (57.03)	15708 (51.17)	27,143 (47.79)	
Intermittent drinker	35,851 (43.22)	21,417 (49.39)	6007 (44.71)		21,708 (41.54)	14143 (46.07)	27,424 (48.28)	
Daily drinker	1593 (1.92)	1535 (3.54)	697 (5.19)		747 (1.43)	846 (2.76)	2232 (3.93)	
SBP, mm Hg	116.16 ± 11.73	130.75 ± 12.3	146.66 ± 16.25	<0.0001	111.83 ± 10.23	123.55 ± 10.36	134.52 ± 14.96	<0.0001
DBP, mm Hg	72.67 ± 8.2	82.72 ± 8.34	90.37 ± 11.06	<0.0001	70.64 ± 7.87	76.13 ± 7.55	84.53 ± 9.63	<0.0001
Fasting Glucose, mg/dL	93.47 ± 22.22	98.08 ± 25.49	103.91 ± 32.39	<0.0001	91.79 ± 20.37	96.33 ± 24.8	99.46 ± 27.4	<0.0001
Total cholesterol, mg/dL	192.03 ± 35.65	200.21 ± 36.33	204.12 ± 38.42	<0.0001	189.15 ± 34.95	196.95 ± 36.3	201.14 ± 36.87	<0.0001
AST, mg/dL	24.6 ± 17.94	27.32 ± 23.45	29.21 ± 22.55	<0.0001	23.7 ± 14.59	26.14 ± 22.45	27.77 ± 23.26	<0.0001
ALT, mg/dL	24.12 ± 22.32	29 ± 28.98	30.15 ± 26.87	<0.0001	22.58 ± 20.05	26.75 ± 25.53	29.27 ± 28.5	<0.0001
**2007–2015 KNAHES Cohort**	**<130 and <80 (*n* = 20,903)**	**130/80–139/89 (*n* = 9983)**	**≥140/90 (*n* = 6949)**	***p*-value ^1^**	**<120 and <80 (*n* = 17,989)**	**120–129 and <80 (*n* = 2914)**	**≥130/80 (*n* = 16,932)**	***p*-value ^1^**
Age	48.4 ± 12.5	52.0 ± 11.9	55.4 ± 11.6	<0.0001	46.765 ± 11.9	58.469 ± 11.709	53.439 ± 11.933	<0.0001
Female Sex, N (%)	13704 (65.56)	4714 (47.22)	3175 (45.69)	<0.0001	11,986 (66.63)	1718 (58.96)	7889 (46.59)	<0.0001
BMI, kg/m^2^	23.2 ± 3.1	24.5 ± 3.3	25.1 ± 3.3	<0.0001	5623 (32.20)	1039 (36.96)	7504 (45.87)	<0.0001
Physical activity, N (%)	1233 (6.15)	603 (6.31)	430 (6.46)	00.6341	2266 (6.25)	1080 (6.25)	153 (5.52)	0.2307
Household income, N (%)				<0.0001				<0.0001
Q1	2988 (14.49)	1654 (16.83)	1616 (23.63)		2199 (12.39)	789 (27.51)	3270 (19.62)	
Q2	5277 (25.60)	2518 (25.63)	1829 (26.74)		4464(25.15)	813 (28.35)	4347 (26.08)	
Q3	6128 (29.72)	2735 (27.83)	1764 (25.79)		5415 (30.51)	713 (24.86)	4499 (27.00)	
Q4	6224 (30.19)	2919 (29.71)	1630 (23.83)		5671 (31.95)	553 (19.28)	4549 (27.30)	
Smokers, N (%)	6662 (32.86)	4361 (45.19)	3143 (46.85)	<0.0001	7573 (45.48)	978 (37.94)	7085 (48.50)	<0.0001
Alcohol drinkers, N (%)	8551 (44.46)	4234 (48.50)	2851 (48.51)	<0.0001	23.072 ± 3.072	24.238 ± 3.151	24.751 ± 3.295	<0.0001
SBP, mm Hg	108.22 ± 9.639	124.22 ± 8.702	143.71 ± 14.043	<0.0001	105.71 ± 7.833	123.71 ± 2.93	132.22 ± 14.75	<0.0001
DBP, mm Hg	69.876 ± 6.256	81.892 ± 4.81	90.245 ± 9.73	<0.0001	69.367 ± 6.233	73.017 ± 5.429	85.32 ± 8.329	<0.0001
Pulse rate	57.557 ± 12.041	57.992 ± 12.185	60.412 ± 16.426	0.0002	57.531 ± 11.812	57.659 ± 12.921	59.042 ± 14.225	0.0217
Fasting Glucose, mg/dL	96.55 ± 21.92	101.61 ± 24.799	104.42 ± 25.985	<0.0001	95.269 ± 20.484	104.52 ± 28.035	102.76 ± 25.328	<0.0001
HbA1c, mg/dL	5.827 ± 0.946	5.981 ± 1.005	6.12 ± 1.081	<0.0001	5.765 ± 0.897	6.17 ± 1.124	6.036 ± 1.038	<0.0001
Insulin, mg/dL	9.209 ± 6.171	10.146 ± 6.963	10.575 ± 7.307	<0.0001	9.03 ± 5.71	10.361 ± 8.48	10.328 ± 7.114	<0.0001
Total cholesterol, mg/dL	187.28 ± 34.381	195.58 ± 35.902	199.46 ± 38.069	<0.0001	186.61 ± 34.123	191.4 ± 35.675	197.17 ± 36.854	<0.0001
Triglycerides, mg/dL	120.61 ± 88.481	154.95 ± 124.52	174.44 ± 142.33	<0.0001	116.98 ± 86.061	143.13 ± 99.322	162.93 ± 132.45	<0.0001
HDL-cholesterol, mg/dL	50.173 ± 11.786	48.489 ± 11.6	48.216 ± 11.583	<0.0001	50.481 ± 11.828	48.248 ± 11.331	48.377 ± 11.594	<0.0001
LDL-cholesterol, mg/dL	113.09 ± 31.369	116.23 ± 34.847	116.34 ± 38.254	<0.0001	112.82 ± 30.927	114.73 ± 33.952	116.28 ± 36.283	<0.0001
AST, mg/dL	21.302 ± 11.932	24.121 ± 14.125	25.489 ± 14.695	<0.0001	20.962 ± 11.371	23.416 ± 14.781	24.681 ± 14.376	<0.0001
ALT, mg/dL	19.931 ± 18.982	24.604 ± 18.695	25.672 ± 17.601	<0.0001	19.613 ± 18.845	21.904 ± 19.698	25.042 ± 18.262	<0.0001
BUN, mg/dL	14.144 ± 4.162	14.783 ± 4.362	15.098 ± 4.576	<0.0001	13.953 ± 4.065	15.326 ± 4.545	14.912 ± 4.454	<0.0001
Creatinine, mg/dL	0.812 ± 0.228	0.865 ± 0.259	0.869 ± 0.283	<0.0001	0.808 ± 0.219	0.837 ± 0.276	0.867 ± 0.269	<0.0001
WBC, mg/dL	5.951 ± 1.693	6.263 ± 1.761	6.423 ± 1.781	<0.0001	5.915 ± 1.685	6.175 ± 1.73	6.328 ± 1.771	<0.0001

Data are expressed as the mean ± SD or frequency (percentage). ^1^ *p*-values were calculated using one-way ANOVA, Chi-square test, and post-hoc analysis. Abbreviations: JNC7, the Joint National Committee 7; 2017 ACC/AHA, 2017 American College of Cardiology/American Heart Association; SBP, Systolic blood pressure; DBP, diastolic blood pressure; AST, Aspartate transaminase; ALT, Alanine aminotransferase; BUN, Blood Urea Nitrogen; WBC, White blood cell.

**Table 2:** Warranty periods for all-cause mortality, all cardiovascular death, and major cardiovascular death according to JNC7 and 2017 ACC/AHA guidelines.

Guidelines			2006–2015 NHIS-HEALS					2007–2015 KNAHES Cohorts				
		Groups	Warranty Period (0.5%)	*n*	Person-Time (Years)	Events, N (%)	Incidence per 1000 Person-Years (95% CI)	Warranty Period (0.5%)	*n*	Person-Time (Years)	Events, N (%)	Incidence per 1000 Person-Years (95% CI)
JNC7	ACM	<130 and <80	3.59	84,653	784,456.07	1854 (2.19)	2.36 (0.15–4.57)	2.45	20,903	11,2374.2	352 (1.68)	3.13 (2.81–3.46)
		130/80–139/89	2.42	44,214	407,805.67	1548 (3.50)	3.80 (0.73–6.86)	2.24	9983	54,240.97	220 (2.20)	4.06 (3.52–4.60)
		≥140/90	1.24	13,751	123,886.58	1209 (8.79)	9.76 (4.22–15.30)	1.72	6949	38,376.21	226 (3.25)	5.89 (5.12–6.66)
	ACD	<130 and <80	9.67	84,653	784,456.07	254 (0.30)	0.32 (0.00–2.54)	8.19	20,903	11,2374.2	60 (0.29)	0.53 (0.40–0.67)
		130/80–139/89	8.42	44,214	407,805.67	279 (0.63)	0.68 (0.00–3.75)	6.28	9983	54,240.97	38 (0.38)	0.70 (0.48–0.92)
		≥140/90	2.75	13,751	123,886.58	286 (2.08)	2.31 (0.00–7.87)	3.5	6949	38,376.21	57 (0.82)	1.49 (1.10–1.87)
	MACE	<130 and <80	9.68	84,653	784,456.07	120 (0.14)	0.15 (0.00–2.37)	9.5	20,903	11,2374.2	32 (0.15)	0.29 (0.19–0.38)
		130/80–139/89	9.84	44,214	407,805.67	136 (0.31)	0.33 (0.00–3.40)	9.5	9983	54,240.97	19 (0.19)	0.35 (0.19–0.51)
		≥140/90	4.08	13,751	123,886.58	150 (1.09)	1.21 (0.00–6.78)	6.5	6949	38,376.21	29 (0.42)	0.76 (0.48–1.03)
2017 ACC/AHA	ACM	<120 and <80	3.51	53,374	494,529.82	1051 (1.97)	2.13 (0.00–4.91)	2.74	17,989	97,368.52	259 (1.44)	2.66 (2.34–2.98)
		120–129 and <80	3.67	31,279	289,926.25	803 (2.57)	2.77 (0.00–6.41)	1.72	2914	15,005.72	93 (3.19)	6.20 (4.94–7.45)
		≥130/80	1.83	57,965	531,692.25	2757 (4.76)	5.19 (2.50–7.87)	1.92	16,932	92,617.18	446 (2.63)	4.82 (4.37–5.26)
	ACD	<120 and <80	9.51	53,374	494,529.82	123 (0.23)	0.25 (0.00–3.04)	8.21	17,989	97,368.52	35(0.19)	0.36 (0.24–0.48)
		120–129 and <80	6.59	31,279	289,926.25	131 (0.42)	0.45 (0.00–4.09)	3.28	2914	15,005.72	25 (0.86)	1.67 (1.01–2.32)
		≥130/80	6.17	57,965	531,692.25	565 (0.97)	1.06 (0.00–3.75)	4.78	16,932	92,617.18	95 (0.56)	1.03 (0.82–1.23)
	MACE	<120 and <80	8.76	53,374	494,529.82	57 (0.11)	0.12 (0.00–2.90)	9.5	17,989	97,368.52	18 (0.10)	0.19 (0.10–0.27)
		120–129 and <80	9.68	31,279	289,926.25	63 (0.20)	0.22 (0.00–3.86)	4.91	2914	15,005.72	14 (0.48)	0.93 (0.44–1.42)
		≥130/80	9.34	57,965	531,692.25	286 (0.49)	0.54 (0.00–3.23)	8.16	16,932	92,617.18	48 (0.28)	0.51 (0.37–0.67)

Abbreviations: JNC7, the Joint National Committee 7; 2017 ACC/AHA, 2017 American College of Cardiology/American Heart Association; ACM: All-cause mortality; ACD: All cardiovascular death; MACE: Major cardiovascular death; CI: Confidence interval.

**Table 3:** Hazard ratios for all-cause mortality, all cardiovascular death, and major cardiovascular death according to JNC7 and 2017 ACC/AHA guidelines.

**Guidelines**			**2006–2015 NHIS-HEALS**				
		**Groups**	**Adjusted HR (95% CI) ^1^**	***p*-Value for Trend**	**Pairwise Comparison *p*-Value**		
JNC7	ACM	<130 and <80	1	<0.0001	ref		
		130/80–139/89	1.10 (1.02–1.18)		0.0102	ref	
		≥140/90	1.84 (1.70–2.00)		<0.0001	<0.0001	ref
	ACD	<130 and <80	1	<0.0001	ref		
		130/80–139/89	1.36 (1.13–1.62)		0.0011	ref	
		≥140/90	2.72 (2.26–3.27)		<0.0001	<0.0001	Ref
	MACE	<130 and <80	1	<0.0001	ref		
		130/80–139/89	1.40 (1.08–1.81)		0.012	ref	
		≥140/90	3.25 (2.51–4.21)		<0.0001	<0.0001	Ref
2017 ACC/AHA	ACM	<120 and <80	1	<0.0001	ref		
		120–129 and <80	0.77 (0.69–0.84)		<0.0001	ref	
		≥130/80	1.15 (1.06–1.24)		0.0005	<0.0001	Ref
	ACD	<120 and <80	1	<0.0001	ref		
		120–129 and <80	0.99 (0.77–1.28)		0.9098	ref	
		≥130/80	1.76 (1.42–2.17)		<0.0001	<0.0001	Ref
	MACE	<120 and <80	1	<0.0001	ref		
		120–129 and <80	1.14 (0.79–1.65)		0.4968	ref	
		≥130/80	2.09 (1.53–2.84)		<0.0001	<0.0001	Ref
**Guidelines**			**2007–2015 KNAHES Cohorts**				
		**Groups**	**Adjusted HR (95% CI) ^2^**	***p* for Trend**	**Pairwise Comparison *p*-Value**		
JNC7	ACM	<130 and <80	1	0.0234	ref.		
		130/80–139/89	0.97 (0.82–1.15)		0.7352	ref.	
		≥140/90	1.24 (1.05–1.46)		0.0128	0.0095	ref.
	ACD	<130 and <80	1	0.0022	ref.		
		130/80–139/89	1.00 (0.66–1.51)		0.9963	ref.	
		≥140/90	1.83 (1.26–2.65)		0.0015	0.0041	ref.
	MACE	<130 and <80	1	0.0482	ref.		
		130/80–139/89	0.91 (0.51–1.62)		0.7520	ref.	
		≥140/90	1.73 (1.04–2.90)		0.0361	0.0298	ref.
2017 ACC/AHA	ACM	<120 and <80	1	0.0592	ref.		
		120–129 and <80	1.37 (1.08–1.74)		0.0101	ref.	
		≥130/80	1.18(1.01–1.38)		0.0407	0.1913	ref.
	ACD	<120 and <80	1	0.0051	ref.		
		120–129 and <80	2.59 (1.54–4.36)		0.0003	ref.	
		≥130/80	1.86 (1.25–2.77)		0.0022	0.1446	ref.
	MACE	<120 and <80	1	0.0654	ref.		
		120–129 and <80	2.88 (1.42–5.87)		0.0034	ref.	
		≥130/80	1.81 (1.04–3.15)		0.0363	0.1271	ref.

**^1^** Adjusted for age, sex, BMI, physical activity, household income, smoking status, alcohol status, fasting serum glucose, and total cholesterol. **^2^** Adjusted for age, sex, BMI. ACM: All-cause mortality; ACD: All cardiovascular death; MACE: Major cardiovascular death; HR: Hazard ratio; CI: Confidence interval.

**Table 4:** Discrimination ability for all-cause mortality, all cardiovascular death, and major cardiovascular death according to JNC7 and 2017 ACC/AHA guidelines.

	2006–2015 NHIS-HEALS			2007–2015 KNAHES Cohort		
	JNC 7	2017 ACC/AHA	*p*-Value	JNC 7	2017 ACC/AHA	*p*-Value
ACM	0.622 (0.613–0.63)	0.605 (0.597–0.612)	<0.0001	0.566 (0.546–0.584)	0.57 (0.551–0.586)	0.4237
ACD	0.682 (0.666–0.704)	0.656 (0.643–0.673)	<0.0001	0.607 (0.561–0.649)	0.613 (0.575–0.645)	0.5854
MACE	0.692 (0.667–0.716)	0.663 (0.644–0.686)	<0.0001	0.582 (0.533–0.636)	0.596 (0.544–0.639)	0.3861

JNC7, the Joint National Committee 7; 2017 ACC/AHA, 2017 American College of Cardiology/American Heart Association; ACM: All-cause mortality; ACD: All cardiovascular death; MACE: Major cardiovascular death. *p*-values were calculated using Harrell’s c-index.
